# Neuromodulatory Effect of Thymoquinone in Attenuating Glutamate-Mediated Neurotoxicity Targeting the Amyloidogenic and Apoptotic Pathways

**DOI:** 10.3389/fneur.2018.00236

**Published:** 2018-04-13

**Authors:** Ibram Amin Fouad, Nadia Mohamed Sharaf, Ragwa Mansour Abdelghany, Nesrine Salah El Dine El Sayed

**Affiliations:** ^1^Department of Pharmacology and Toxicology, German University in Cairo, New Cairo, Egypt; ^2^Department of Pharmacology and Toxicology, Faculty of Pharmacy, Cairo University, Cairo, Egypt

**Keywords:** amyloid-beta, caspase-3, cytochrome *c*, excitotoxicity, glutamate, thymoquinone

## Abstract

Overexposure of the glutamatergic N-methyl-d-aspartate (NMDA) receptor to the excitatory neurotransmitter l-glutamic acid leads to neuronal cell death by excitotoxicity as a result of increased intracellular Ca^2+^, mitochondrial dysfunction, and apoptosis. Moreover, it was previously reported that prolonged activation of the NMDA receptor increased beta-amyloid (Aβ) levels in the brain. Thymoquinone (TQ), the active constituent of *Nigella sativa* seeds, has been shown to have potent antioxidant and antiapoptotic effects. The aim of the present study was to explore the neuromodulatory effects of different doses of TQ (2.5 and 10 mg/kg) against apoptotic cell death and Aβ formation resulting from glutamate administration in rats using vitamin E as a positive control. Behavioral changes were assessed using Y-maze and Morris water maze tests for evaluating spatial memory and cognitive functions. Caspase-3, Lactate dehydrogenase, Aβ-42, and cytochrome *c* gene expression were determined. TQ-treated groups showed significant decreases in the levels of all tested biochemical and behavioral parameters compared with the glutamate-treated group. These findings demonstrated that TQ has a promising neuroprotective activity against glutamate-induced neurotoxicity and this effect is mediated through its anti-amyloidogenic, antioxidant, and antiapoptotic activities.

## Introduction

Glutamate (Glu) is the primary excitatory neurotransmitter in the brain which contributes to many physiological processes, including learning and memory (1). Pathological overstimulation of glutamatergic receptors produces an excessive influx of Ca^+2^ and Na^+^, leading to glutamate-induced neuronal apoptosis and eliciting a marked excitotoxicity (2). Over-activation of the glutamatergic N-methyl-d-aspartate (NMDA) receptor in the brain provokes excitotoxic neuronal death which plays a crucial role in many pathological conditions, including ischemic stroke, traumatic brain injury, Alzheimer’s disease (AD), Parkinson’s disease (PD), and epilepsy (3). Overactivation of NMDA receptors by Glu is associated with apoptotic cell death *via* release of cytochrome *c* (Cyto-*c*) and induction of the intrinsic apoptotic pathway (4, 5). Cyto-*c* initiates the caspase-dependent apoptotic pathway, which causes proteolytic processing of pro-Caspase-9 and subsequently activation of the downstream effectors caspase-3 (Casp-3), -6, and -7 (6). Casp-3 causes degradation of nuclear DNA (7) and formation of peroxynitrite radicals (ONOO^−^), provoking depletion of cellular energy (8). Moreover, NMDA overactivation was found to increase the production of amyloid-beta (Aβ) protein *via* different potential mechanisms: (1) Ca^2+^-dependent shift from non-amyloidogenic to amyloidogenic processing of the amyloid precursor protein (APP) (9–11) and impaired clearance of the Aβ protein from brain and blood (12, 13) and (2) alteration of proteolysis of APP by Casp-3 leading to an increase in Aβ-42 peptide levels (14, 15).

Owing to its antioxidant property, thymoquinone (TQ) was proven to protect different organs against pathological conditions caused by oxidative damage. TQ was also reported as a potent neuroprotective agent against neurodegeneration induced by forebrain ischemia by attenuating oxidative stress (16). Its antioxidant activity may be attributable to the effect of its reduced form tert-butylhydroquinone, which acts as a hydrogen donating antioxidant that inhibits lipid peroxidation, or to the scavenging effect of multiple reactive oxygen species by TQ and thymohydroquinone, mimicking superoxide dismutase activity (17). Moreover, TQ can restore the abnormal matrix metalloproteinase and hence decrease reactive oxygen species levels (18). *In vitro*, TQ had been found to counteract the oxidative stress and membrane potential collapse induced by Aβ-42 aggregation (19). TQ was found to protect against hepatic apoptosis induced by ischemia reperfusion injury (20, 21). It was also proven to be an effective antineoplastic agent by triggering cancer apoptosis and autophagic cell death (22), as well as suppressing tumor angiogenesis (23). TQ also exerted an anti-inflammatory effect against rheumatoid arthritis (24) and bronchial asthma (25). Moreover, TQ demonstrated potential anticonvulsant activity against petit-mal epilepsy (26).

Vitamin E (Vit E) is one of the most powerful natural antioxidants and is essential for protecting the body from free radical damage (27). It is considered to be a potent protective agent against atherosclerosis, AD, and cancer (28). Moreover, Vit E was found to protect against apoptotic cell death induced by cinnamaldehyde in PLC/PRF/5 cells (29), ultra-violet B radiations in chicken embryonic fibroblasts (30), and haloperidol in hippocampal cell lines (31).

The aim of the present study was to explore the neuromodulatory effect of different doses of TQ 2.5 and 10 mg/kg against glutamate-induced neuronal damage, by assessing the behavioral changes (spatial memory and cognitive function) and biochemical parameters [Cyto-*c*, Casp-3, lactate dehydrogenase (LDH), and Aβ levels] in an animal model using Vit E as a positive control.

## Materials and Methods

### Material

#### Experimental Animals

Adult male albino rats (weight: 250–300 g; age: ~2 months) were used. The animals were obtained from the animal colony of the National Institute of Research (Cairo, Egypt). The rats were housed in a temperature-controlled room (23–24°C) with a 12-h light:dark cycle and with free access to food and water. They were allowed to acclimatize to the animal house of the German University in Cairo for at least 1 week before initiating the experiments. Animal procedures were performed following the approval of the Ethics Committee of the German University in Cairo in association with the recommendations of the National Institutes of Health Guide for Care and Use of Laboratory Animals (Publication No. 85-23, revised 1985). All efforts were made to minimize animal discomfort and suffering.

#### Drugs and Chemicals

l-Glutamic acid monosodium hydrate, TQ, and Vit E were purchased from Sigma-Aldrich Co., USA. Saline 0.9% (NaCl), 70% ethanol, and olive oil were purchased from Adwic (El-Nasr Pharmaceutical Chemicals Co., Egypt), and phosphate buffered saline was purchased from Lonza Ltd., Switzerland.

#### Experimental Design

Two control groups were analyzed in the present study. The first group received 1 ml of 0.9% saline i.p. daily for 14 consecutive days and the second group received 1 ml of olive oil i.p. daily for 14 consecutive days. No statistically significant differences were noted in the results among the two control groups, thus, they were pooled into a single control group referred to as the Negative control group in the experimental design and the description of results.

Seventy-two rats weighting were used in the present study and were allocated to five groups, of 12 rats each.

Group I was the Negative control group, while rats in Group II were injected with Glu (2 g/kg, i.p.) once daily for seven consecutive days (1). Group III was referred to as the VitE/Glu Group in which rats were injected with Vit E (50 mg/kg, i.p.) once daily for seven consecutive days followed by glutamate (2 g/kg, i.p.) for an additional seven consecutive days (32). In Group IV; the TQ2.5/Glu Group, rats received TQ (2.5 mg/kg, i.p.) once daily for seven consecutive days, followed by glutamate (2 g/kg i.p.) for an additional seven consecutive days (33). The same protocol was applied in Group 5 (the TQ10/Glu Group) although the dose of TQ was increased to 10 mg/kg (33).

### Methods

#### Neurobehavioral Tests

On the last day of drug treatment, animals were trained for the Y-maze and day 1 of the Morris water maze (MWM). The following day, animals were tested for the Y-maze test and day 2 of MWM. Then, the third and fourth trials of MWM as well as the probe test were conducted on three successive days. Every apparatus was thoroughly washed with 70% ethanol between each use and after every animal in both the training and testing sessions (34).

##### Y-Maze

The Y-maze is used to measure spatial working memory in rodents *via* the spontaneous alternation behavior (SAB) calculation (35). Spontaneous alternation measures the ability of the animal to alternate its choice of arm entry on subsequent trials based on its memory of pervious arm entries performed (35), which depends on the natural exploratory behavior of rats for new environments (36). The maze is a Y-shaped apparatus consisting of three arms, each with the same dimensions, 35-cm long, 25-cm high, and 10-cm wide, and each extending from a central platform, with the angle between each arm equal 120°. The apparatus was placed on the floor of the experimental room. The test was performed for 2 days; the first day was for the purpose of training, during which each rat was positioned in the central platform and allowed to explore the maze freely for 8 min. The same procedure was followed on the second testing day, with the addition of manual recording of each arm entry, scored only when all four limbs of the rat were inside the arm. After each session the maze was cleaned with 70% ethanol to exclude any olfactory cues that might interfere with subsequent testing. An alternation was considered to have occurred if three successive different arms were entered during an overlapping triplet set. The percentage of spontaneous alteration activity (SAB%) was calculated as the “number of consecutive alternations” divided by “the total number of arms entries minus 2” and multiplied by 100 (37).

##### Morris Water Maze

The MWM is one of the most frequently applied *in vivo* neurobehavioral tests in neuroscience (38). Its advantage over other neurobehavioral tests is its ability to assess and differentiate deficits in memory formation from other types of deficits unrelated to memory including sensory, motor, motivational, and retrieval processes (39). The test is swimming based model employing distal clues, in which the animal swims from a starting location and navigates to a hidden submerged escape platform. The swimming environment is a large, circular stainless steel pool (150 cm in diameter, 60 cm in height, maintained at a temperature of 25 ± 1°C) half filled with water and divided into four quadrant using two threads positioned perpendicular to each other on the edges of the pool. The platform is positioned 2 cm below the water level in the target quadrant. The platform position remained unchanged throughout the training session, and was rendered invisible by coloring the water using a non-toxic dye. Visual clues, such as colored shapes and stickers, were placed around the pool in plain sight of the animal. The position of the experimenter was constant so as not to disturb the relative location of the water maze with respect to other objects in the laboratory, which serve as prominent visual cues. For four consecutive days, each rat was subjected to two trials where, for each trial, the rat was allowed to search for the fixed platform for 120 s. The gap between the two trials was 10–15 min. On the fifth day, the platform was removed, and each rat was subjected to a probe test during which it was required to swim for 60 s. The time spent by the animal within the probe (target) quadrant was recorded (the quadrant that held the platform during the training days). Time spent in the target quadrant is considered to be an index of memory retrieval (40–42).

#### Biochemical Assessments

##### Tissue Sampling

The rats were sacrificed by cervical dislocation and decapitation, after which, brains were divided into two equal hemispheres in an ice: salt mixture. Each hemisphere was homogenized with the appropriate buffer, according to the assay kits described below.

##### Detection of Cyto-*c* Gene Expression in Brain Tissue

Briefly, total RNA was extracted using the Qiagen tissue extraction kit (Qiagen, USA) from the rat brain tissue samples. RNA concentration was obtained by spectrophotometry (Dual wavelength spectrophotometer, Beckman, USA) and was reverse transcribed to cDNA with a high capacity cDNA reverse transcription kit (Fermentas, USA). The qPCR assay was carried out in which amplification and analysis of the converted cDNA were processed employing an Applied Biosystem with software version 3.1. The qPCR assay was optimized by adjusting the annealing temperature such that the sample, together with the primer sets and SYBR Green I, were able to bind. A typical qPCR run includes roughly 40 cycles. The Ct of a sample is defined as the number of PCR cycles necessary for the fluorescent signal produced by SYBR Green to cross above a threshold in the linear part of the amplification curve and it is employed in the analysis step. Relative quantification (RQ), using GAPDH gene expression as an endogenous control, was performed for all samples tested. The RQ was calculated using the 2-DCCT (2^−ΔΔCt^) method (43).

##### Determination of Casp-3 Levels in Brain Tissue

Caspase 3 levels in the brain samples were assayed using Casp-3 ELISA kit (Cusabio, China). This assay measures the amount of sample by sandwiching it between two antibodies one of which is pre-coated to the microtiter plate and the other of which acts as a detector antibody (44). Antibodies specific for Casp-3 were precoated onto a microplate. Standards and samples are pipetted into the wells and any Casp-3 present is bound by the immobilized antibody. After removing any unbound substances, a biotin-conjugated antibody specific for Casp-3 is then added to the wells. After washing, avidin conjugated horseradish peroxidase (HRP) is added to the wells. Following a wash to remove any unbound avidin-enzyme reagent, a substrate solution is added to the wells and color develops in proportion to the amount of Casp-3 bound in the initial step. The color development is stopped and the intensity of the color is measured. Casp-3 levels in the brain were expressed as nanograms/milliliter and calculated from a constructed standard curve using known concentrations of Casp-3.

##### Determination of LDH in Brain Tissue

Lactate dehydrogenase levels in the brain samples were assayed using an LDH ELISA kit (Stanbio Lab., USA). The microtiter plate provided in this kit was precoated with an antibody specific to LDH. Standards or samples are then added to the appropriate microtiter plate wells with a biotin-conjugated polyclonal antibody preparation specific for LDH and avidin conjugated to HRP is added to each microplate well and incubated. Then, a TMB (3,3′,5,5′ tetramethyl-benzidine) substrate solution is added to each well. Only those wells that contain LDH, biotin-conjugated antibody and enzyme-conjugated avidin will exhibit a change in color. The enzyme–substrate reaction is terminated by the addition of a sulfuric acid solution and the color change is measured spectrophotometrically at a wavelength of 450 ± 2 nm. LDH levels in the brain were expressed as units/liter and calculated from a constructed standard curve using known concentrations of LDH.

##### Determination of Amyloid-Beta in Brain Tissue

Aβ1–42 levels in the brain samples were assayed using an Aβ-42 ELISA kit (MyBiosource, USA). The assay measures the amount of sample by sandwiching it between two antibodies one of which is precoated to the microtiter plate and the other of which acts as a detector antibody (44). The microtiter plate provided in this kit was precoated with an antibody specific to Aβ1–42. Standards or samples are then added to the appropriate microtiter plate wells with a biotin-conjugated antibody preparation specific for Aβ1–42 and avidin conjugated to HRP is added to each microplate well and incubated. Then, a TMB substrate solution is added to each well. Only those wells that contain Aβ1–42, biotin-conjugated antibody and enzyme-conjugated avidin will exhibit a change in color. The enzyme–substrate reaction is terminated by addition of a sulfuric acid solution and the color change is measured spectrophotometrically at a wave length of 450 ± 2 nm. Brain Aβ (1–42) level was expressed as picograms/milliliter and calculated from a constructed standard curve using known concentration of Aβ (1–42).

### Statistical Analysis

Data were represented as mean ± SE. Results were analyzed by one-way ANOVA followed by Tukey’s Multiple Comparison Test, by the aid of Graph pad prism 5 software. *p*-Value (*p* < 0.0 5) was considered significant. In the present study *, @, #, and X were utilized to denote statistical difference from different groups according to the following scheme: *: statistically significant from negative control group by *p* value (*p* < 0.0 5). @: statistically significant from Glu group by *p* value (*p* < 0.0 5). #: statistically significant from Vit E/Glu group by *p* value (*p* < 0.0 5). X: statistically significant from TQ 2.5/Glu group by *p* value (*p* < 0.0 5).

Results of TQ10/Glu Group were compared to that ofTQ 2.5/Glu, VitE/Glu, and Glu Group. Results of VitE/Glu group were compared to that of Glu group. Results of Glu group were compared to negative control group.

## Results

### Neurobehavioral Tests

#### Effect of Different Doses of TQ and Vit E on Spatial Memory in the Y-Maze Test

**Figure 1:**
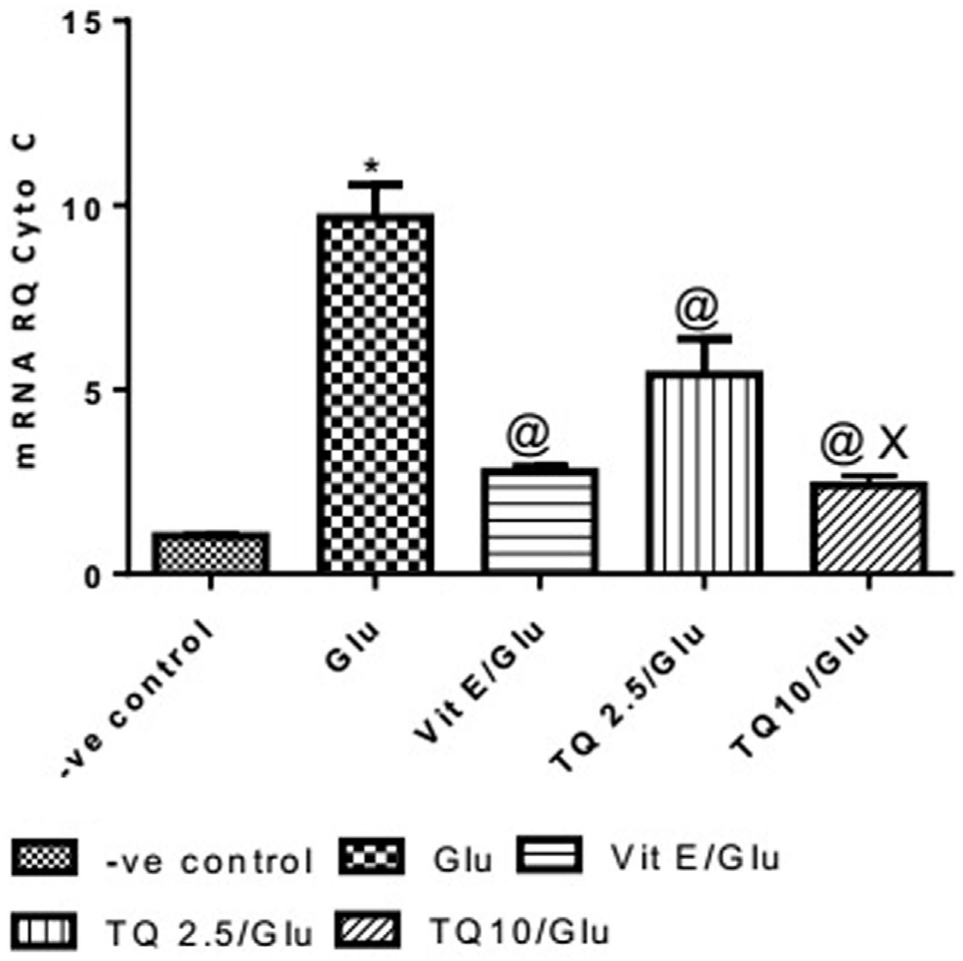
Effect of Glu, Vit E/Glu, TQ2.5/Glu, and TQ10/Glu on spatial memory in the Y-maze test. Glu was injected i.p. as a single dose for 7 days. TQ and Vit E were injected as a single dose for 7 days. Each value represents the mean value of 12 rats ± SEM. Statistics were carried out by one-way ANOVA followed by Tukey’s multiple comparison tests, by graph pad prism 5. *Statistically significant from −ve control group (*p* < 0.05); ^@^statistically significant from Glu group (*p* < 0.05); ^#^statistically significant from Vit E/Glu group (*p* < 0.05); ^X^statistically significant from TQ 2.5/Glu group (*p* < 0.05).

Glu group showed significant decreased in the SAB% when compared to negative control group by 51.9%. Vit E group showed significant improvement in SAB% when was compared to Glu group by 116%. TQ (2.5 and 10 mg/kg) showed a significant increase in SAB% when compared to Glu group by 109.5 and 112.6%, respectively (Table 1, Figure 1).

**Table 1:** Effects of Glu, Vit E/Glu, TQ 2.5/Glu, and TQ 10/Glu on the spatial memory in the Y-maze test.

Groups	−ve control	Glu	Vit E/Glu	TQ 2.5/Glu	TQ 10/Glu
SAB%	75.92 ± 8.200	36.53 ± 7.609*	78.85 ± 6.99^@^	76.50 ± 4.15^@^	77.60 ± 4.885^@^

*Effect of Glu, Vit E/Glu, TQ2.5/Glu, and TQ10/Glu on spatial memory. Glu was injected i.p. as a single dose for 7 days. TQ and Vit E were injected as a single dose for 7 days. Animals were subjected to the Y-maze test and the SAB% for each rat was recorded for 8 min and the mean SAB% for all rats in each group was calculated. Statistical analysis of mean SAB% were carried out by one-way ANOVA followed by Tukey’s multiple comparison tests, by graph pad prism 5. Each value represent the mean value of 12 rats ± SEM*.

*Statistically significant from −ve control group (p < 0.05).

*^@^Statistically significant from Glu group (p < 0.05)*.

#### Effect of Different Doses of TQ and Vit E on the Mean Escape Latency (MEL) in the MWM Test

**Figure 2:**
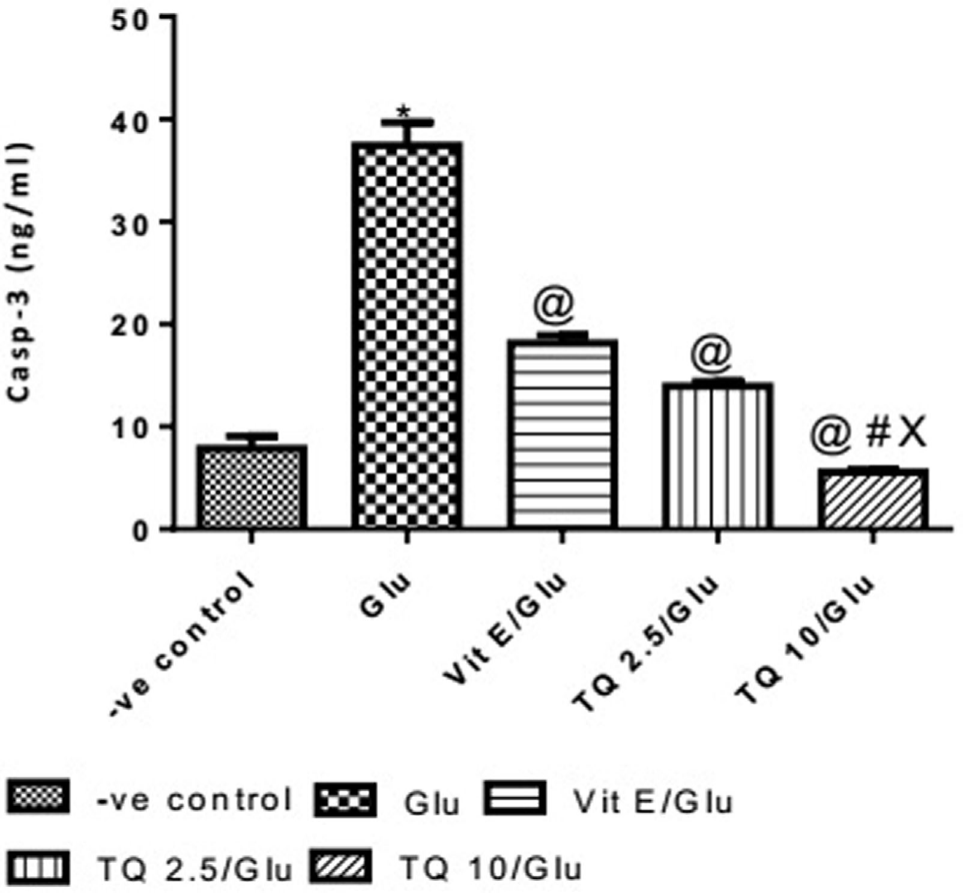
Effect of Glu, Vit E/Glu, TQ2.5/Glu, and TQ10/Glu on the fourth day mean escape latency in the Morris water maze test. Glu was injected i.p. as a single dose for 7 days. TQ and Vit E were injected as a single dose for 7 days. Each value represents the mean value of 12 rats ± SEM. Statistics carried out by one-way ANOVA followed by Tukey’s multiple comparison tests, by graph pad prism 5. *Statistically significant from −ve control group (*p* < 0.05); ^@^statistically significant from Glu group (*p* < 0.05); ^#^statistically significant from Vit E/Glu group (*p* < 0.05); ^X^statistically significant from TQ 2.5/Glu group (*p* < 0.05).

**Figure 3:**
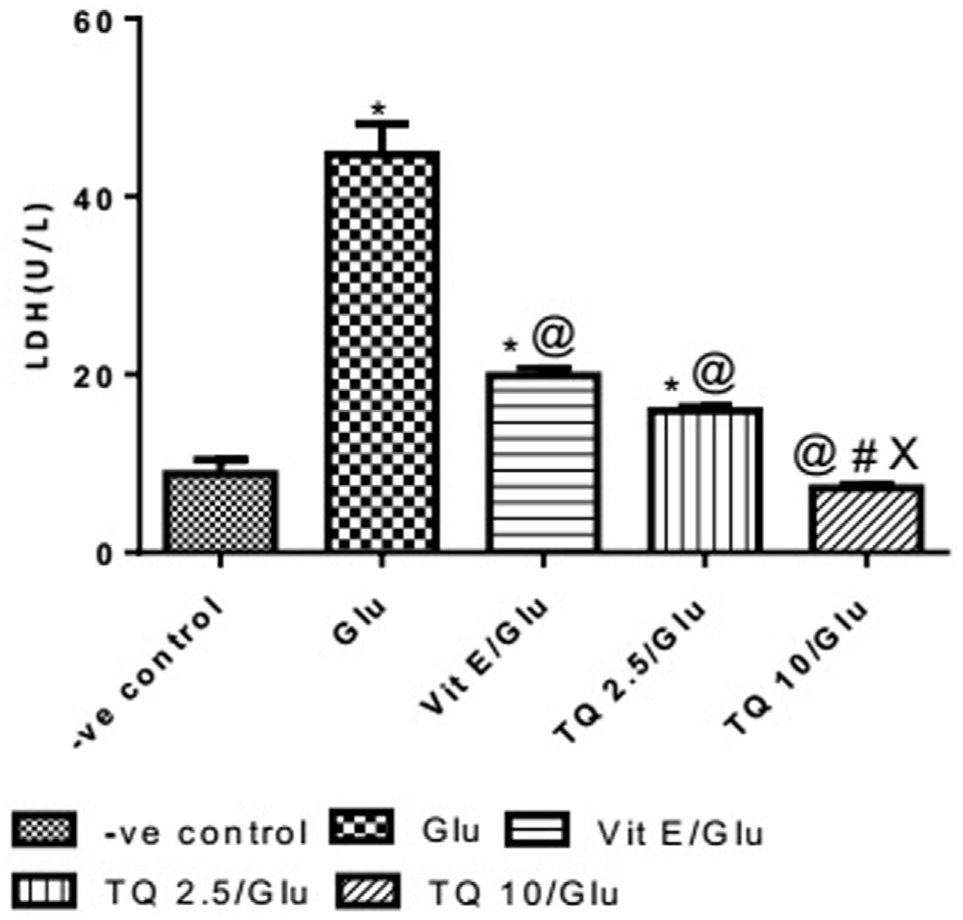
Effect of Glu, Vit E/Glu, TQ2.5/Glu, and TQ10/Glu on mean escape latency in the Morris water maze test. Glu was injected i.p. as a single dose for 7 days. TQ and Vit E were injected as a single dose for 7 days. Each value represents the mean value of 12 rats ± SEM. Statistics were carried out by one-way ANOVA followed by Tukey’s multiple comparison tests, by graph pad prism 5. *Statistically significant from −ve control group (*p* < 0.05); ^@^statistically significant from Glu group (*p* < 0.05); ^#^statistically significant from Vit E/Glu group (*p* < 0.05); ^X^statistically significant from TQ 2.5/Glu group (*p* < 0.05).

The effect of TQ andVit E was estimated on the MEL using MWM after four testing days. MEL on the fourth day was recorded for each mouse. Glu showed significant increase in MEL when compared to the negative control group by 110.12%. Vit E caused a significant reduction in MEL when compared to Glu group by 70.5%. TQ 2.5 and 10 mg/kg showed a significant decrease in MEL when compared to Glu group by 39.4 and 46.3%, respectively (Table 2, Figures 2, 3).

**Table 2:** Effect of Glu, Vit E/Glu, TQ 2.5/Glu, and TQ 10/Glu on the mean escape latency (MEL) in Morris water maze (MWM) test.

Days	−ve control	Glu	Vit E/Glu	TQ 2.5/Glu	TQ 10/Glu
1st Day MEL (s)	24.46 ± 4.377	62.666670 ± 14.92	44.42 ± 18.88	61.75 ± 10.20	32.58 ± 5.943
2nd Day MEL (s)	23.54 ± 2.208	63.833330 ± 16.55	16.15 ± 3.975	17.08 ± 2.336	23.42 ± 10.03
3rd Day MEL (s)	19.96 ± 2.792	47.683330 ± 9.323	17.80 ± 4.245	12.92 ± 1.67	19.5 ± 5.583
4th Day MEL (s)	11.46 ± 2.208	24.083340 ± 5.352*	7.80 ± 1.913^@^	14.58 ± 3.197^@^	12.92 ± 3.446^@^

*Effect of Glu, Vit E/Glu, TQ2.5/Glu, and TQ10/Glu on the fourth day MEL in MWM. Glu was injected i.p. as a single dose for 7 days. TQ and Vit E were injected as a single dose for 7 days. Animals in each group were subjected to MWM test. Animals were subjected to eight training trials, two trials sessions each day for four consecutive days with an inter-trial interval of 5–15 min and the MEL of each animal was recorded for 2 min in each trial. The average MEL of each animal was recorded for the four consecutive days and statistical analysis of the mean MEL of animals in each group were carried out by one-way ANOVA followed by Tukey’s multiple comparison tests, by graph pad prism 5. Each value represent the mean value of 12 rats ± SEM. Significant difference between the groups in MEL onthe fourth day was recorded*.

*Statistically significant from −ve control group (*p* < 0.05).

*^@^Statistically significant from Glu group (*p* < 0.05)*.

#### Effect of Different Doses of TQ and Vit E on the Probe Test in the MWM Test

**Figure 4:**
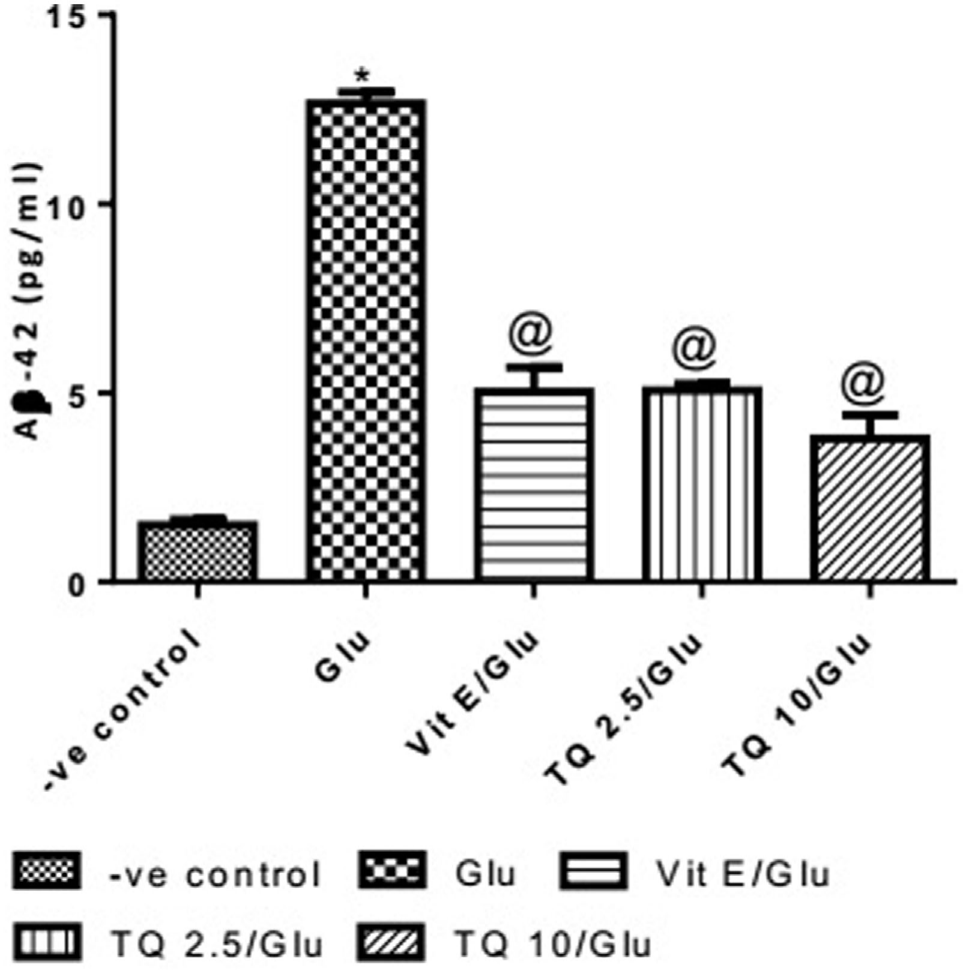
Effect of Glu, Vit E/Glu, TQ2.5/Glu, and TQ10/Glu on the probe test in Morris water maze. Glu was injected i.p. as a single dose for 7 days. TQ and Vit E were injected as a single dose for 7 days. Each value represents the mean value of 12 rats ± SEM. Statistics were carried out by one-way ANOVA followed by Tukey’s multiple comparison tests, by graph pad prism 5. *Statistically significant from −ve control group (*p* < 0.05); ^@^statistically significant from Glu group (*p* < 0.05); ^#^statistically significant from Vit E/Glu group (*p* < 0.05); ^X^statistically significant from TQ 2.5/Glu group (*p* < 0.05).

The time spent in the target quadrant of the MWM was evaluated by the probe test. Glu group showed a significant decrease in the time spent in the target quadrant when compared to the negative control group by 64%. Vit E group significantly increased the time spent in target quadrant when compared to Glu group by 120.5%. TQ (2.5 and 10 mg/kg) showed a significant increase in the time spent in the target quadrant when compared to Glu group by 114.7 and 117.6%, respectively (Table 3, Figure 4).

**Table 3:** Effect of Glu, Vit E/Glu, TQ 2.5/Glu, and TQ 10/Glu on the probe test in Morris water maze (MWM).

Groups	−ve control	Glu	Vit E/Glu	TQ 2.5/Glu	TQ 10/Glu
Mean time spent in the target quadrant (s)	23.75 ± 0.9320	8.500 ± 1.118*	18.75 ± 1.726^@^	18.25 ± 1.078^@^	18.5 ±1.4232^@^

*Glu was injected i.p. as a single dose for 7 days. TQ and Vit E were injected as a single dose for 7 days. Animals in each group were subjected to MWM test. Animals were subjected to probe test and time spent in the target quadrant of each rat was recorded for 60-s period on the fifth day of MWM test. The mean time spent in the target quadrant of all rats in each group was calculated and expressed in seconds. Statistical analysis of mean time spent in target quadrant were carried out by one-way ANOVA followed by Tukey’s multiple comparison tests, by graph pad prism 5*.

*Statistically significant from −ve control group (*p* < 0.05).

*^@^Statistically significant from Glu group (*p* < 0.05)*.

### Biochemical Parameters

#### Effect of Different Doses of TQ and Vit E on the Cyto-*c* Gene Expression in Brain Tissue

**Figure 5:**
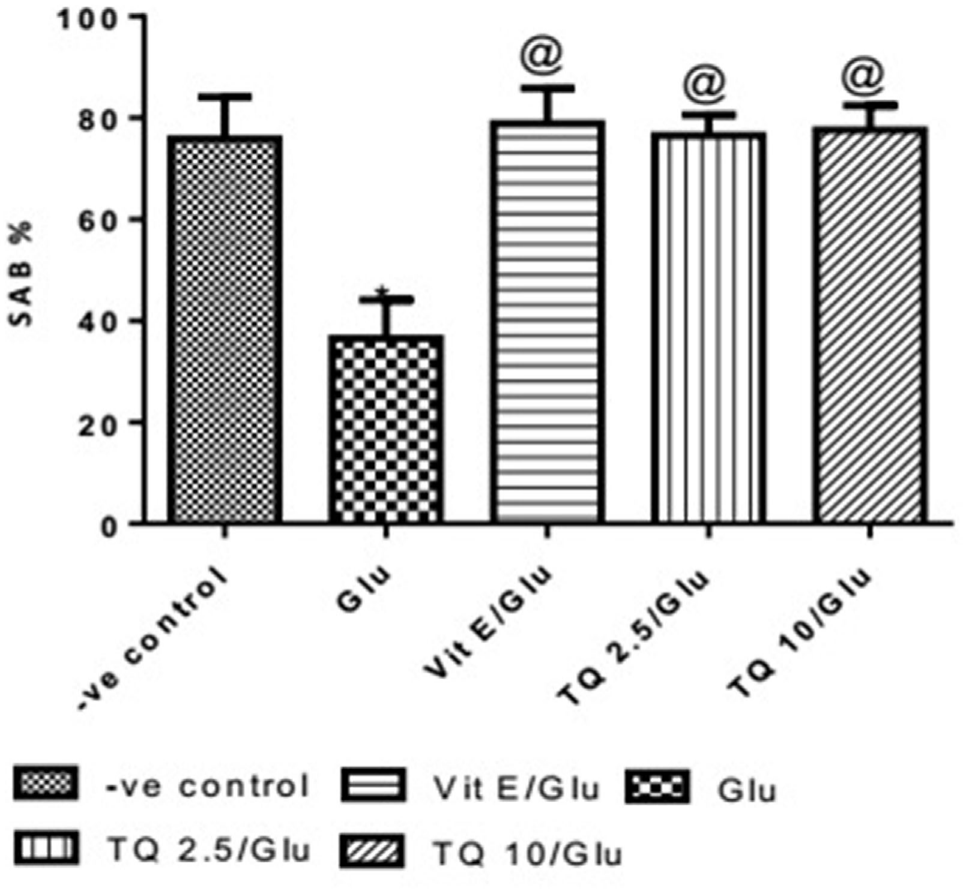
Effect of Glu, Vit E/Glu, TQ2.5/Glu, and TQ10/Glu on Cyto-C gene expression. Glu was injected i.p. as a single dose for 7 days. TQ and Vit E were injected as a single dose for 7 days. Each value represents the mean value of 12 rats ± SEM. Statistics were carried out by one-way ANOVA followed by Tukey’s multiple comparison tests, by graph pad prism 5. *statistically significant from −ve control group (*p* < 0.05); ^@^statistically significant from Glu group (*p* < 0.05); ^#^statistically significant from Vit E/Glu group (*p* < 0.05); ^X^statistically significant from TQ 2.5/Glu group (*p* < 0.05).

Glu group showed a significant increase in the expression of Cyto-*c* gene, when compared to negative control by 837.01%. Vit E group significantly reduced the expression of Cyto-*c* gene, when compared to Glu group by 71.35%. TQ (2.5 and 10 mg/kg) produced a significant decrease in the expression of Cyto-*c* gene when compared to Glu group by 44.08 and 75.18%, respectively. TQ (10 mg/kg) reduced significantly the expression of Cyto-*c* gene when compared to Vit E group by 14.98%; moreover, it showed a significant decrease when compared to TQ (2.5 mg/kg) by 55.64% (Table 4, Figure 5).

**Table 4:** Effect of Glu, Vit E/Glu, TQ2.5/Glu, and TQ10/Glu on Cyto-*c* gene expression.

Groups	−ve control	Chi	Vit E/Glu	TQ 2.5/Glu	TQ 10/Glu
Cyto-*c* gene expression	1.032 ± 0.014	9.67 ± 0.895*	2.77 ± 0.148^@^	5.407 ± 0.98^@^	2.40 ± 0.264^X^^,^^@^

*Glu was injected i.p. as a single dose for 7 days. TQ and Vit E were injected as a single dose for 7 days. Animals in each group were subjected to the neurobehavioral tests. Once they were scarified, their brains were retrieved and homogenized in its suitable buffer and used in RT-PCR assay*.

*Statistical analysis of mean Cyto-*c* gene expression (RQ) were performed using one-way analysis of variance (ANOVA) followed by Tukey’s multiple comparison tests, by graph pad prism 5. Each value represents the mean value of 12 rats ± SEM*.

*Statistically significant from −ve control group (*p* < 0.05).

*^@^Statistically significant from Glu group (*p* < 0.05)*.

*^X^Statistically significant from TQ 2.5/Glu group (*p* < 0.05)*.

#### Effect of Different Doses of TQ and Vit E on the Casp-3 Levels in Brain Tissue

**Figure 6:**
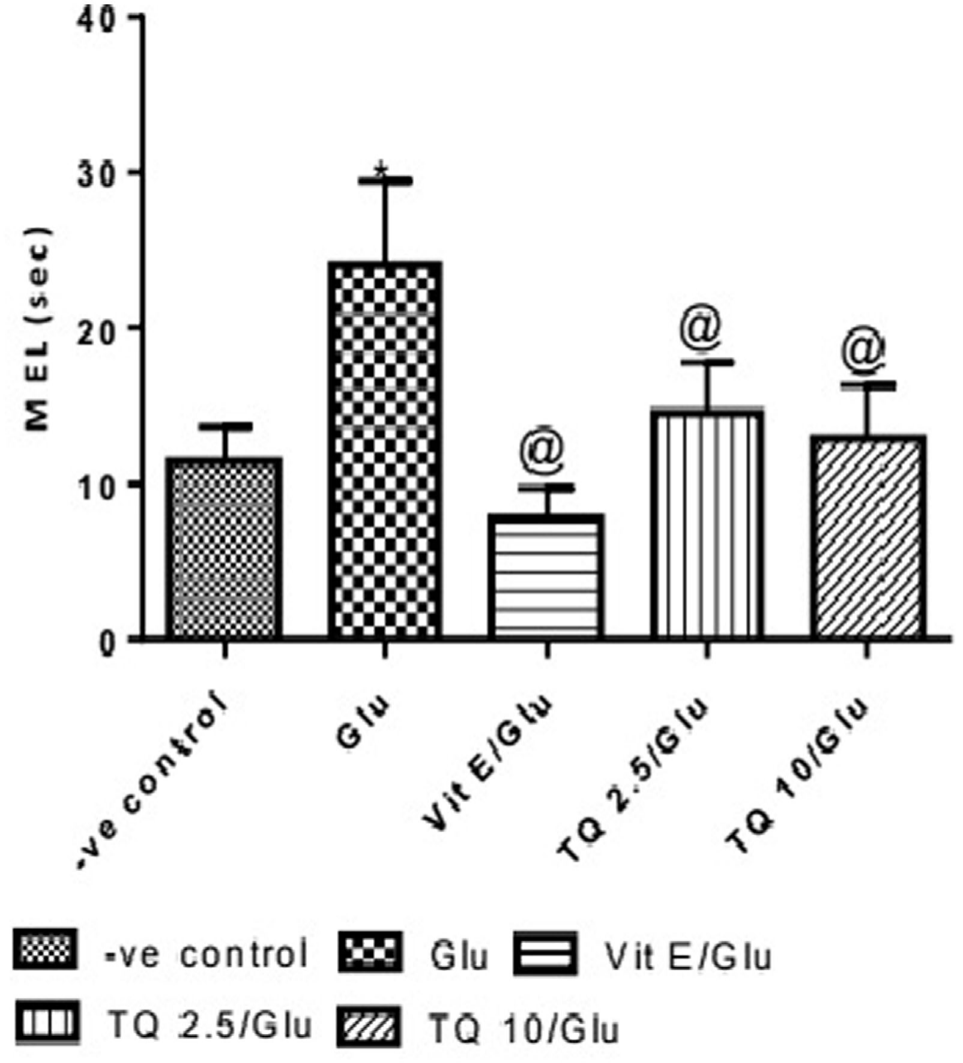
Effect of −ve control, Glu, Vit E/Glu, TQ2.5/Glu, and TQ10/Glu on Casp-3 levels. Glu was injected i.p. as a single dose for 7 days. TQ and Vit E were injected as a single dose for 7 days. Each value represents the mean value of 12 rats ± SEM. Statistics were carried out by one-way ANOVA followed by Tukey’s multiple comparison tests, by graph pad prism 5. *statistically significant from −ve control group (*p* < 0.05); ^@^statistically significant from Glu group (*p* < 0.05); ^#^statistically significant from Vit E/Glu group (*p* < 0.05); ^X^statistically significant from TQ 2.5/Glu group (*p* < 0.05).

The Glu group showed a significant increase in the levels of Casp-3 when compared to negative control group by 378%. Vit E group showed a significant decrease in the level of Casp-3 when compared to Glu group by 51.51%. TQ (2.5 and 10 mg/kg) produced a significant decrease in the level of Casp-3 when compared to Glu Group by 62.74 and 85.17%, respectively. TQ (2.5 mg/kg group) reduced the level of Casp-3, compared to Vit E group by 23.14%. TQ (10 mg/kg) group showed a significant decrease in the level of Casp-3 when compared to Vit E and TQ 2.5/kg groups by 69.44 and 60.22%, respectively (Table 5, Figure 6).

**Table 5:** Effects of Glu, Vit E/Glu, TQ 2.5/Glu, and TQ 10/Glu on Casp-3 level.

Group	−ve control	Glu	Vit E/Glu	TQ 2.5/Glu	TQ 10/Glu
Concentration of Caspase-3 (ng/ml)	7.833 ± 1.218	37.43 ± 2.260*	18.15 ± 0.7610^@^	13.95 ± 0.4288^@^	5.550 ± 0.2742^@^^,^^#^^,^^X^

*Glu was injected i.p. as a single dose for 7 days. TQ and Vit E were injected as a single dose for 7 days. Animals in each group were subjected to the neurobehavioral tests before being sacrificed. The brains were isolated and homogenized as 10% homogenate in 0.1 M PBS then centrifuged, and supernatants were used in the Casp-3 ELISA kit. Statistical analyses of mean Casp-3 level were performed using ANOVA followed by Tukey’s multiple comparison tests, by graph pad prism 5. Each value represent the mean value of 12 rats ± SEM*.

*Statistically significant from −ve control group (*p* < 0.05).

*^@^Statistically significant from Glu group (*p* < 0.05)*.

*^#^Statistically significant from Vit E/Glu group (*p* < 0.05)*.

*^X^Statistically significant from TQ 2.5/Glu group (*p* < 0.05)*.

#### Effect of Different Doses of TQ and Vit E on the LDH Levels in Brain Tissue

**Figure 7:**
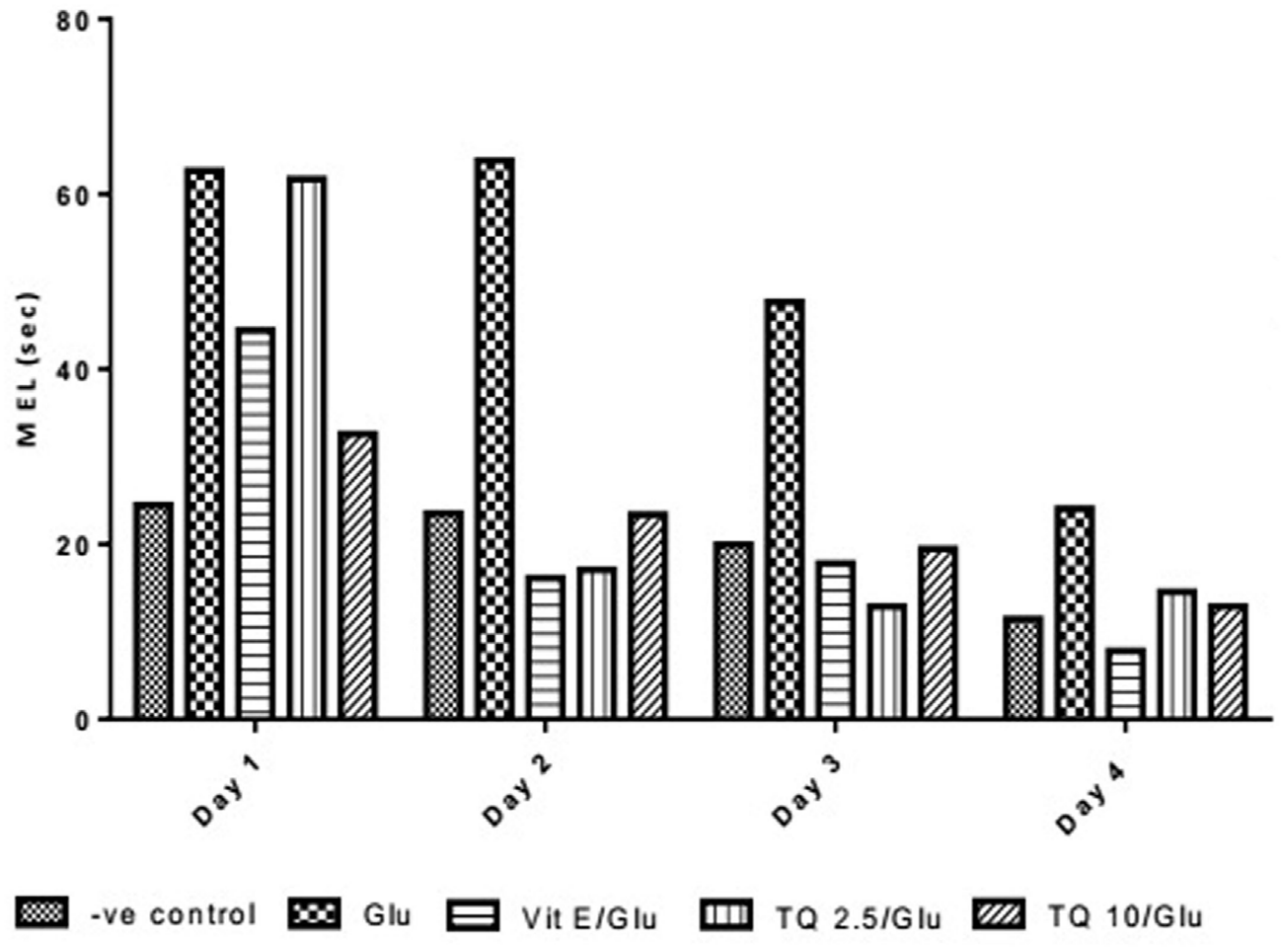
Effect of Glu, Vit E/Glu, TQ2.5/Glu, and TQ10/Glu on lactate dehydrogenase levels. Glu was injected i.p. as a single dose for 7 days. TQ and Vit E were injected as a single dose for 7 days. Each value represents the mean value of 12 rats ± SEM. Statistics were carried out by one-way ANOVA followed by Tukey’s multiple comparison tests, by graph pad prism 5. *Statistically significant from −ve control group (*p* < 0.05); ^@^statistically significant from Glu group (*p* < 0.05); ^#^statistically significant from Vit E/Glu group (*p* < 0.05); ^X^statistically significant from TQ 2.5/Glu group (*p* < 0.05).

Glu group showed significant increase in the level of LDH when was compared to −ve control by 453.04%. Vit E group showed significant decrease in the level of LDH when compared to Glu group by 55.5%. TQ 2.5 and 10 mg/kg groups showed a significant reduction in the level of LDH when compared to Glu group by 64.40 and 83.40%, respectively. TQ 2.5 mg/kg group showed decrease in LDH level when compared to Vit E group by 19.91%. TQ (10 mg/kg) significantly reduced the LDH level than Vit E and TQ 2.5 mg/kg groups by 62.64 and 53.42%, respectively (Table 6, Figure 7).

**Table 6:** Effect of −ve control, Glu, Vit E/Glu, TQ 2.5/Glu, and TQ 10/Glu on lactate dehydrogenase (LDH) level.

Groups	−ve control	Glu	Vit E/Glu	TQ 2.5/Glu	TQ 10/Glu
Mean level of LDH (U/l)	8.088 ± 1.593	44.73 ± 3.418*	19.89 ± 0.8119^@^	1S.93 ± 0.4335^@^	7.423 ± 0.3897^@^^,^^#^^,^^X^

*Glu was injected i.p. as a single dose for 7 days. TQ and Vit E were injected as a single dose for 7 days. Animals were subjected to the neurobehavioral tests before being sacrificed. The brains were isolated and homogenized as 10% homogenate in 0.1 M PBS then centrifuged, and supernatants were used in the LDH ELISA kit. Statistical analyses of mean LDH level were performed using ANOVA followed by Tukey’s multiple comparison tests, by graph pad prism 5. Each value represents the mean value of 12 rats ± SEM*.

*Statistically significant from −ve control group (*p* < 0.05).

*^@^Statistically significant from Glu group (*p* < 0.05)*.

*^#^Statistically significant from Vit E/Glu group (*p* < 0.05)*.

*^X^Statistically significant from TQ 2.5/Glu group (*p* < 0.05)*.

#### Effect of Different Doses of TQ and Vit E on the Aβ-42 Levels in Brain Tissue

**Figure 8:**
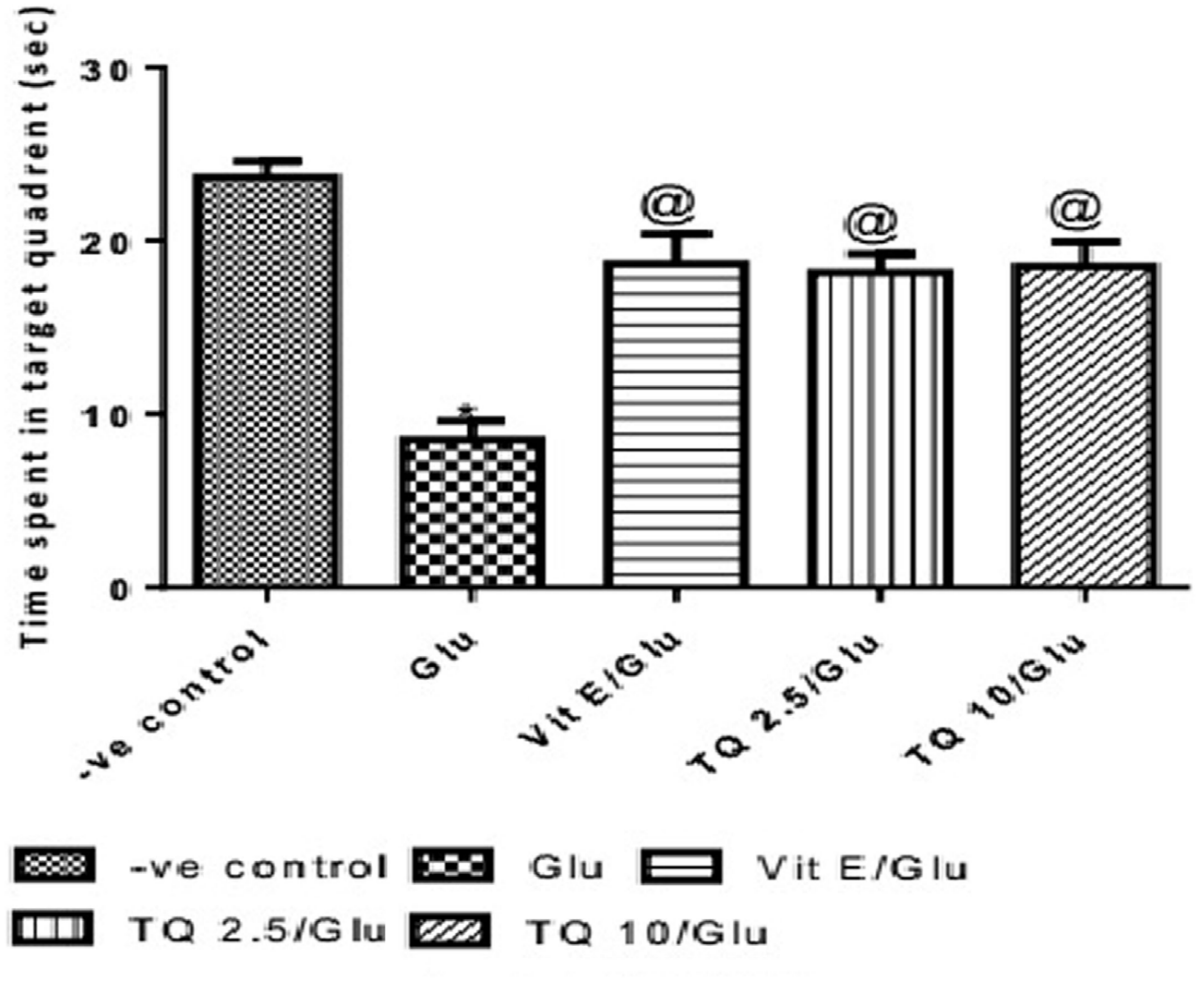
Effect of −ve control, Glu, Vit E/Glu, TQ2.5/Glu, and TQ10/Glu on Aβ(1–42) levels. Glu was injected i.p. as a single dose for 7 days. TQ and Vit E were injected as a single dose for 7 days. Each value represents the mean value of 12 rats ± SEM. Statistics were carried out by one-way ANOVA followed by Tukey’s multiple comparison tests, by graph pad prism 5. *Statistically significant from −ve control group (*p* < 0.05); ^@^statistically significant from Glu group (*p* < 0.05); ^#^statistically significant from Vit E/Glu group (*p* < 0.05); ^X^statistically significant from TQ 2.5/Glu group (*p* < 0.05).

Glu produced a significant increase in the level of Aβ-42 protein when compared to negative control group by 743.33%. Vit E significantly reduced the Aβ1–42 level when compared to Glu group by 60.21%. TQ (2.5 and 10 mg/kg) showed a significant decrease in the Aβ-42 levels when compared to Glu group by 59.60 and 70.04%, respectively (Table 7, Figure 8).

**Table 7:** Effect of Glu, Vit E/Glu, TQ 2.5/Glu, and TQ 10/Glu on the Aβ-42 levels.

Groups	−ve control	Glu	Vit E/Glu	TQ 2.5/Glu	TQ 10/Glu
Aβ-42 pg/ml	1.500 ± 0.155	12.65 ± 0.312*	5.033 ± 0.644^@^	5.107 ± 0.150^@^	3.793 ± 0.622^@^

*Glu was injected i.p. as a single dose for 7 days. TQ and Vit E were injected as a single dose for 7 days. Animals in each group were subjected to the neurobehavioral tests before being sacrificed. The brains were isolated and homogenized as 10% homogenate in 0.1 M PBS then centrifuged, and supernatants were used in the Aβ-42 ELISA kit. Statistical analyses of mean Aβ-42 level were performed using ANOVA followed by Tukey’s multiple comparison tests, by graph pad prism 5. Each value represents the mean value of 12 rats ± SEM*.

*Statistically significant from −ve control group (*p* < 0.05).

*^@^Statistically significant from Glu group (*p* < 0.05)*.

## Discussion

Overactivation of NMDA receptors by Glu induces neuronal apoptosis through excitotoxicity and contributes to many neurological disorders (45). Importantly, overactivation of NMDA receptors by glutamate also leads to an increase in the level of Aβ1–42, by shifting the APP processing toward the amyloidogenic pathway, resulting in subsequent cognitive dysfunction (10, 46).

In the current study, administration of glutamate produced a significant alteration in spatial memory, as measured by the Y-maze test. This coincides with previous studies whereby Glu-excitotoxicity induced by several agents caused alteration in the SAB%, compared to control groups, in the Y-maze test (47–49). Previously, it was shown that bilateral intracerebroventricular administration of Aβ-35 caused impairment in the SAB% and contributed to the neurotoxicity of Aβ through increasing glutamatergic neurotransmission (49). Moreover, it was observed that olfactory bulbectomy in rodents led to deficits in behavioral and cognitive deficits through glutamate-mediated excitotoxicity manifested by impairment in the SAB% (50). Furthermore, NMDA receptor overactivation during ischemia in mice contributed to apoptotic cell death in neuronal cells leading to behavioral impairment, also manifested by a decrease in SAB% in the Y-maze test (51). The MWM is a common test used to assess cognitive and behavioral impairment induced by the administration of monosodium glutamate, which serves as a traumatic brain injury model. Glutamate administration caused spatial reference memory deficits, spatial discrimination deficits and cognitive dysfunction, as shown by an increase in MEL and a decrease in time spent in the target quadrant; these deficits can be attributed to hippocampal cell death induced by excitotoxicity (52–56). Consistent with these findings, the current study confirmed that Glu administration for seven consecutive days in the Glu group caused impairment in the spatial memory function which was evidenced by a decrease in MEL and also in the time spent in target quadrant. These findings could be attributed to the increase in the apoptotic cell death markers (Cyto-*c*, Casp-3, and LDH) observed in the present study. This coincides with previous studies showing that either acute or repeated administration of Glu induced the intrinsic pathway of apoptotic cell death through the release of Cyto-*c* and induction of caspase-dependent cell death evidenced by an increase in Cyto-*c* and Casp-3 levels (57–59). It was reported the main role of Glu-excitotoxicity in spatial memory impairment induction is through apoptotic cell death, which was confirmed by the increase in caspase activity and demonstrated by the MWM test (60). Taken together, these studies support the view suggested by the current study that the Glu-excitotoxicity contribution to spatial memory impairment occurs mainly through apoptotic neuronal cell death as a downstream mechanism, which was also suggested by the observed increase in the levels of LDH.

In the current study, Glu administration in the Glu group resulted in an increase in Aβ 1–42 concentration. The mechanism by which excitotoxicity increased Aβ production was previously reported by a study which showed that excitotoxicity following acute brain injury caused an increase in the amyloidogenic processing of APP toward the production of the Aβ protein (14). This finding is in agreement with a previous report revealing that NMDA receptor activation after acute brain injury may significantly contribute to the development of amyloid plaques (10). Moreover, a recent study showed that monosodium glutamate administration induced an increase in Aβ accumulation in the rat hippocampus and induced neurobehavioral abnormalities demonstrated by a decrease in SAB% (61), which coincides with the findings in the present study suggesting a role for the Aβ accumulation in inducing the neurobehavioral deficits observed in rats. In addition, it has been shown that Casp-3 is involved in APP complex proteolysis, causing an increase in Aβ peptide formation (14). Moreover, Aβ production and accumulation within neuronal cortices consequently was found to induce neuronal apoptotic cell death (62). Altogether, these findings, together with the findings of the current study, suggest a reciprocal relationship between Aβ production and apoptotic cell death relative to the spatial memory impairment shown here to be a result of Glu administration.

In the current study, administration of both doses of TQ showed a dose-dependent improvement in spatial memory function, as demonstrated by the neurobehavioral tests. Previous studies showed that oral administration of TQ was able to improve spatial memory impairment induced in diabetic rats, as shown by an increase in the SAB% (63–65). Recently, it was shown that TQ produced a shortening of the time latency in the MWM (66) and an increase in the time spent in the target quadrant (67). Moreover, administration of TQ at a concentration of 10 mg/kg in rats was also able to restore the cognitive impairment induced by status epileptics shown by improvements in the time latency and time spent in the target quadrant in the MWM test (68). The current study assumed that the improvement of spatial memory observed is attributed to the inhibition of apoptotic cell death which was evidenced by the significant decrease in the apoptotic markers (Cyto-*c* and Casp-3). A previous study showed that administration of TQ to cultured cortical neurons relieved the apoptotic cell death triggered in fetal alcohol syndrome by increasing the expression of Bcl-2, leading to inhibition of Cyto-*c* release and suppressing the activity of the apoptotic caspases, including Casp-3 (20). In addition, TQ protected against apoptotic cell death following ischemia reperfusion injury in hepatic cells owing to its antioxidant property, reducing NF-κB and Bcl-2 expression, which was reflected by a decrease in the apoptotic cell markers (21). Moreover, oral administration of TQ attenuated apoptosis of the hippocampus following chronic toluene exposure in rats *via* its antioxidant property, as evidenced by suppression of the activity of Casp-3 (69). Furthermore, the antiapoptotic property of TQ in *Nigella sativa* contributed to the modulation of neuronal cell death in pentylenetetrazol-induced kindling which occurs in part due to NMDA receptor activation (70). These findings further support the assumption proposed by the current study, that the maintenance of spatial memory by TQ, demonstrated by the improved SAB%, MEL and time spent in the target quadrant, can be attributed to its antiapoptotic activity, evidenced by the significant decrease in the apoptotic cell markers and the maintenance of cell viability reflected by the decrease in LDH levels. Regarding Aβ, both doses of TQ (5 and 10 mg/kg) reduced Aβ-42 levels. As discussed above, because of the contribution of apoptotic cell death to the increase in Aβ-42 production (14), the antiapoptotic activity of TQ may contribute to the observed decrease in Aβ-levels. Nanoemulsion of TQ was found to reduce Aβ-42 levels through modulating the processing of APP, decreasing β-secretase and γ-secretase levels and increasing the degradation of Aβ-42 (71). In addition, a previous study showed that TQ reduced Aβ aggregation as a result of its properties as an antioxidant and antiapoptotic agent (72). In agreement with these findings, the present study showed that administration of TQ decreased Aβ levels, which could be due to several different mechanisms: (1) the antiapoptotic properties of TQ, (2) shifting APP processing from the amyloidogenic to the non-amyloidogenic pathway, and (3) increasing the clearance of Aβ. Thus, the current study could be extended by evaluating the effect of TQ on the levels of β- and γ-secretase.

In conclusion, TQ treatment caused a decrease in the level of Cyto-*c*, Casp-3, LDH, and Aβ-42 in brain homogenates, thus proving to be a good choice for restoring memory and cognitive deficits induced by Glu-excitotoxicity, as reflected by improvements in SAB%, MEL, and time spent in the target quadrant. We attribute these results to the ability of TQ to counteract the apoptotic cell death and increased Aβ-42 production-induced by Gluowing to its antiapoptotic properties. As a result, TQ reduced cognitive impairment induced by Glu administration and thus it is a promising therapeutic approach against many neurodegenerative diseases that are induced by Glu-excitotoxicity.

## Ethics Statement

Animal procedures were performed after the approval of the ethics committees of German University in Cairo and Cairo university with the recommendations of the National Institutes of Health Guide for Care and Use of Laboratory Animals.

## Author Contributions

All authors listed have made a substantial, direct, and intellectual contribution to the work and approved it for publication.

## Conflict of Interest Statement

The authors declare that the research was conducted in the absence of any commercial or financial relationships that could be construed as a potential conflict of interest.

## References

[1] Nagakannan P Shivasharan BD Thippeswamy BS Veerapur VP. Restoration of brain antioxidant status by hydroalcoholic extract of *Mimusops elengi* flowers in rats treated with monosodium glutamate. J Environ Pathol Toxicol Oncol (2012) 31(3):213–21. 10.1615/JEnvironPatholToxicolOncol.v31.i3.30 23339696

[2] Mao X-Y Cao Y-G Ji Z Zhou H-H Liu Z-Q Sun H-L. Topiramate protects against glutamate excitotoxicity via activating BDNF/TrkB-dependent ERK pathway in rodent hippocampal neurons. Prog Neuropsychopharmacol Biol Psychiatry (2015) 60:11–7. 10.1016/j.pnpbp.2015.01.015 25661849

[3] Li Y Liu Y Peng X Liu W Zhao F Feng D NMDA receptor antagonist attenuates bleomycin-induced acute lung injury. PLoS One (2015) 10(5):e0125873. 10.1371/journal.pone.0125873 25942563 PMC4420245

[4] Stanika RI Winters CA Pivovarova NB Andrews SB. Differential NMDA receptor-dependent calcium loading and mitochondrial dysfunction in CA1 vs. CA3 hippocampal neurons. Neurobiol Dis (2010) 37(2):403–11. 10.1016/j.nbd.2009.10.020 19879359 PMC2818520

[5] Chen T Fei F Jiang X Zhang L Qu Y Huo K Down-regulation of Homer1b/c attenuates glutamate-mediated excitotoxicity through endoplasmic reticulum and mitochondria pathways in rat cortical neurons. Free Radic Biol Med (2012) 52(1):208–17. 10.1016/j.freeradbiomed.2011.10.451 22080088

[6] Giorgi C Baldassari F Bononi A Bonora M De Marchi E Marchi S Mitochondrial Ca(2+) and apoptosis. Cell Calcium (2012) 52(1):36–43. 10.1016/j.ceca.2012.02.008 22480931 PMC3396846

[7] Das A McDowell M Pava MJ Smith JA Reiter RJ Woodward JJ The inhibition of apoptosis by melatonin in VSC4.1 motoneurons exposed to oxidative stress, glutamate excitotoxicity, or TNF-alpha toxicity involves membrane melatonin receptors. J Pineal Res (2010) 48(2):157–69. 10.1111/j.1600-079X.2009.00739.x 20082663 PMC2862889

[8] Yu S-W Wang H Dawson TM Dawson VL. Poly(ADP-ribose) polymerase-1 and apoptosis inducing factor in neurotoxicity. Neurobiol Dis (2003) 14(3):303–17. 10.1016/j.nbd.2003.08.008 14678748

[9] Sung S Yao Y Uryu K Yang H Lee VM-Y Trojanowski JQ Early vitamin E supplementation in young but not aged mice reduces Abeta levels and amyloid deposition in a transgenic model of Alzheimer’s disease. FASEB J (2004) 18(2):323–5. 10.1096/fj.03-0961fje 14656990

[10] Lesné S Ali C Gabriel C Croci N MacKenzie ET Glabe CG NMDA receptor activation inhibits alpha-secretase and promotes neuronal amyloid-beta production. J Neurosci (2005) 25(41):9367–77. 10.1523/JNEUROSCI.0849-05.2005 16221845 PMC6725703

[11] Zhao Y Zhao B Oxidative stress and the pathogenesis of Alzheimer’s disease. Oxid Med Cell Longev (2013) 2013:Article ID 316523 10.1155/2013/316523 PMC3745981 23983897

[12] Nishida Y Ito S Ohtsuki S Yamamoto N Takahashi T Iwata N Depletion of vitamin E increases amyloid β accumulation by decreasing its clearances from brain and blood in a mousemodel of Alzheimer disease. J Biol Chem (2009) 284(48):33400–8. 10.1074/jbc.M109.054056 19679659 PMC2785184

[13] Yui D Nishida Y Nishina T Mogushi K Tajiri M Ishibashi S Enhanced phospholipase A2 group 3 expression by oxidative stress decreases the insulin-degrading enzyme. PLoS One (2015) 10(12):e0143518. 10.1371/journal.pone.0143518 26637123 PMC4670075

[14] Gervais FG Xu D Robertson GS Vaillancourt JP Zhu Y Huang J Involvement of caspases in proteolytic cleavage of Alzheimer’s amyloid-beta precursor protein and amyloidogenic A beta peptide formation. Cell (1999) 97(3):395–406. 10.1016/S0092-8674(00)80748-5 10319819

[15] Stone JR Okonkwo DO Singleton RH Mutlu LK Helm GA Povlishock JT. Caspase-3-mediated cleavage of amyloid precursor protein and formation of amyloid beta peptide in traumatic axonal injury. J Neurotrauma (2002) 19(5):601–14. 10.1089/089771502753754073 12042095

[16] Al-Majed AA Al-Omar FA Nagi MN. Neuroprotective effects of thymoquinone against transient forebrain ischemia in the rat hippocampus. Eur J Pharmacol (2006) 543(1–3):40–7. 10.1016/j.ejphar.2006.05.046 16828080

[17] Badary OA Taha RA Gamal el-Din AM Abdel-Wahab MH Thymoquinone is a potent superoxide anion scavenger. Drug Chem Toxicol (2003) 26(2):87–98. 10.1081/DCT-120020404 12816394

[18] Khan A Vaibhav K Javed H Khan MM Tabassum R Ahmed ME Attenuation of Aβ-induced neurotoxicity by thymoquinone via inhibition of mitochondrial dysfunction and oxidative stress. Mol Cell Biochem (2012) 369(1–2):55–65. 10.1007/s11010-012-1368-x 22752387

[19] Alhebshi AH Gotoh M Suzuki I Thymoquinone protects cultured rat primary neurons against amyloid β-induced neurotoxicity. Biochem Biophys Res Commun (2013) 433(4):362–7. 10.1016/j.bbrc.2012.11.139 23537659

[20] Ullah I Ullah N Naseer MI Lee HY Kim MOK. Neuroprotection with metformin and thymoquinone against ethanol-induced apoptotic neurodegeneration in prenatal rat cortical neurons. BMC Neurosci (2012) 13:11. 10.1186/1471-2202-13-11 22260211 PMC3317821

[21] Abd El-Ghany RM Sharaf NM Kassem LA Mahran LG Heikal OA Thymoquinone triggers anti-apoptotic signaling targeting death ligand and apoptotic regulators in a model of hepatic ischemia reperfusion injury. Drug Discov Ther (2009) 3(6):296–306. 10.1016/j.toxlet.2009.06.686 22495664

[22] Chen M-C Lee N-H Hsu H-H Ho T-J Tu C-C Hsieh DJ-Y Thymoquinone induces caspase-independent, autophagic cell death in CPT-11-resistant LoVo colon cancer via mitochondrial dysfunction and activation of JNK and p38. J Agric Food Chem (2015) 63(5):1540–6. 10.1021/jf5054063 25611974

[23] Yi T Cho S-G Yi Z Pang X Rodriguez M Wang Y Thymoquinone inhibits tumor angiogenesis and tumor growth through suppressing AKT and extracellular signal-regulated kinase signaling pathways. Mol Cancer Ther (2008) 7(7):1789–96. 10.1158/1535-7163.MCT-08-0124 18644991 PMC2587125

[24] Tekeoglu I Dogan A Ediz L Budancamanak M Demirel A. Effects of thymoquinone (volatile oil of black cumin) on rheumatoid arthritis in rat models. Phytother Res (2007) 21(9):895–7. 10.1002/ptr.2143 17562570

[25] El Mezayen R El Gazzar M Nicolls MR Marecki JC Dreskin SC Nomiyama H. Effect of thymoquinone on cyclooxygenase expression and prostaglandin production in a mouse model of allergic airway inflammation. Immunol Lett (2006) 106(1):72–81. 10.1016/j.imlet.2006.04.012 16762422

[26] Hosseinzadeh H Parvardeh S. Anticonvulsant effects of thymoquinone, the major constituent of *Nigella sativa* seeds, in mice. Phytomedicine (2004) 11(1):56–64. 10.1078/0944-7113-00376 14971722

[27] Sylvester PW. Vitamin E and apoptosis. Vitam Horm (2007) 76:329–56. 10.1016/S0083-6729(07)76012-0 17628180

[28] Rizvi S Raza ST Ahmed F Ahmad A Abbas S Mahdi F. The role of vitamin e in human health and some diseases. Sultan Qaboos Univ Med J (2014) 14(2):e157–65. 24790736 PMC3997530

[29] Wu SJ Ng LT Lin CC. Effects of vitamin E on the cinnamaldehyde-induced apoptotic mechanism in human PLC/PRF/5 cells. Clin Exp Pharmacol Physiol (2004) 31(11):770–6. 10.1111/j.1440-1681.2004.04091.x 15566391

[30] Jin DP Li C Cong Y Yang H Zhang WX Guan W Inhibitory effects of vitamin E on UVB-induced apoptosis of chicken embryonic fibroblasts. Cell Biol Int (2011) 35(4):381–9. 10.1042/CBI20090285 21054279

[31] Post A Rücker M Ohl F Uhr M Holsboer F Almeida OFX Mechanisms underlying the protective potential of alpha-tocopherol (vitamin E) against haloperidol-associated neurotoxicity. Neuropsychopharmacology (2002) 26(3):397–407. 10.1016/S0893-133X(01)00364-5 11850154

[32] Patel Manali B Deshpande S Shah G. Evaluation of efficacy of vitamin E and N-acetyl cysteine in gentamicin-induced nephrotoxicity in rats. Ren Fail (2011) 33(3):341–7. 10.3109/0886022X.2011.560987 21401361

[33] Hosseinzadeh H Parvardeh S Asl MN Sadeghnia HR Ziaee T. Effect of thymoquinone and *Nigella sativa* seeds oil on lipid peroxidation level during global cerebral ischemia-reperfusion injury in rat hippocampus. Phytomedicine (2007) 14(9):621–7. 10.1016/j.phymed.2006.12.005 17291733

[34] Burke SN Wallace JL Nematollahi S Uprety AR Barnes CA. Pattern separation deficits may contribute to age-associated recognition impairments. Behav Neurosci (2010) 124(5):559–73. 10.1037/a0020893 20939657 PMC3071152

[35] Detrait E Brohez C Automation of continuous spontaneous alternation to increase the throughput for in vivo screening of cognitive enhancers. Optimization of the ethovision sys. Proc Meas (2010) 2010:141–4.

[36] Wietrzych M Meziane H Sutter A Ghyselinck N Chapman PF Chambon P Working memory deficits in retinoid X receptor γ-deficient mice. Learn Mem (2005) 12(3):318–26. 10.1101/lm.89805 15897255 PMC1142461

[37] Võikar V Vasar E Rauvala H. Behavioral alterations induced by repeated testing in C57BL/6J and 129S2/Sv mice: implications for phenotyping screens. Genes Brain Behav (2004) 3(1):27–38. 10.1046/j.1601-183X.2003.0044.x 14960013

[38] Engelmann M Ebner K Landgraf R Wotjak CT. Effects of Morris water maze testing on the neuroendocrine stress response and intrahypothalamic release of vasopressin and oxytocin in the rat. Horm Behav (2006) 50(3):496–501. 10.1016/j.yhbeh.2006.04.009 16875693

[39] McNamara RK Skelton RW. The neuropharmacological and neurochemical basis of place learning in the Morris water maze. Brain Res Rev (1993) 18(1):33–49. 10.1016/0165-0173(93)90006-L 8467349

[40] Singh B Sharma B Jaggi AS Singh N Attenuating effect of lisinopril and telmisartan in intracerebroventricular streptozotocin induced experimental dementia of Alzheimer’s disease type: possible involvement of PPAR-γ agonistic property. J Renin Angiotensin Aldosterone Syst (2013) 14(2):124–36. 10.1177/1470320312459977 23060470

[41] Blokland A Geraerts E Been M A detailed analysis of rats’ spatial memory in a probe trial of a Morris task. Behav Brain Res (2004) 154(1):71–5. 10.1016/j.bbr.2004.01.022 15302112

[42] Vorhees CV Williams MT. Morris water maze: procedures for assessing spatial and related forms of learning and memory. Nat Protoc (2006) 1(2):848–58. 10.1038/nprot.2006.116 17406317 PMC2895266

[43] Livak KJ Schmittgen TD Analysis of relative gene expression data using real-time quantitative PCR and the 2−ΔΔCT method. Methods (2001) 25(4):402–8. 10.1006/meth.2001.1262 11846609

[44] Engvall E Perlmann P Enzyme-linked immunosorbent assay (ELISA). Quantitative assay of immunoglobulin G. Immunochemistry (1971) 8(9):871–4. 10.1016/0019-2791(71)90454-X 5135623

[45] Mehta A Prabhakar M Kumar P Deshmukh R Sharma PL. Excitotoxicity: bridge to various triggers in neurodegenerative disorders. Eur J Pharmacol (2013) 698(1–3):6–18. 10.1016/j.ejphar.2012.10.032 23123057

[46] Revett TJ Baker GB Jhamandas J Kar S Protective effect of *Nigella sativa* and thymoquinone on serum/glucose deprivation-induced DNA damage in PC12 cells. J Psychiatry Neurosci (2013) 38(1):6–23. 10.22038/ajp.2012.98 22894822 PMC3529221

[47] Mori K Togashi H Ueno KI Matsumoto M Yoshioka M. Aminoguanidine prevented the impairment of learning behavior and hippocampal long-term potentiation following transient cerebral ischemia. Behav Brain Res (2001) 120(2):159–68. 10.1016/S0166-4328(00)00371-5 11182164

[48] Dillon GM Qu X Marcus JN Dodart J-C. Excitotoxic lesions restricted to the dorsal CA1 field of the hippocampus impair spatial memory and extinction learning in C57BL/6 mice. Neurobiol Learn Mem (2008) 90(2):426–33. 10.1016/j.nlm.2008.05.008 18602845

[49] Yenkoyan K Safaryan K Navasardyan G Mkrtchyan L Aghajanov M Effects of beta-amyloid on behavioral and amino acids spectrum in rats’ brain and their modulation by embryonic proteins. Neurochem Int (2009) 54(5–6):292–8. 10.1016/j.neuint.2008.12.010 19121356

[50] Douma TN Borre Y Hendriksen H Olivier B Oosting RS. Simvastatin improves learning and memory in control but not in olfactory bulbectomized rats. Psychopharmacology (Berl) (2011) 216(4):537–44. 10.1007/s00213-011-2245-0 21384104 PMC3140942

[51] Gigler G Szénási G Simó A Lévay G Hársing LG Sas K Neuroprotective effect of L-kynurenine sulfate administered before focal cerebral ischemia in mice and global cerebral ischemia in gerbils. Eur J Pharmacol (2007) 564(1–3):116–22. 10.1016/j.ejphar.2007.02.029 17407777

[52] Fisher KN Turner RA Pineault G Kleim J Saari MJ. The postweaning housing environment determines expression of learning deficit associated with neonatal monosodium glutamate (M.S.G.). Neurotoxicol Teratol (1991) 13(5):507–13. 10.1016/0892-0362(91)90058-5 1758404

[53] Hamm RJ O’Dell DM Pike BR Lyeth BG. Cognitive impairment following traumatic brain injury: the effect of pre- and post-injury administration of scopolamine and MK-801. Brain Res Cogn Brain Res (1993) 1(4):223–6. 10.1016/0926-6410(93)90006-Q 8003921

[54] Wong PT Neo LH Teo WL Feng H Xue YD Loke WH. Deficits in water escape performance and alterations in hippocampal cholinergic mechanisms associated with neonatal monosodium glutamate treatment in mice. Pharmacol Biochem Behav (1997) 57(1–2):383–8. 10.1016/S0091-3057(96)00338-3 9164598

[55] Xu Y Yan J Zhou P Li J Gao H Xia Y Neurotransmitter receptors and cognitive dysfunction in Alzheimer’s disease and Parkinson’s disease. Prog Neurobiol (2012) 97(1):1–13. 10.1016/j.pneurobio.2012.02.002 22387368 PMC3371373

[56] Abu-Taweel GM Zyadah MA Ajarem JS Ahmad M. Cognitive and biochemical effects of monosodium glutamate and aspartame, administered individually and in combination in male albino mice. Neurotoxicol Teratol (2014) 42:60–7. 10.1016/j.ntt.2014.02.001 24556450

[57] Ness JK Scaduto RC Wood TL. IGF-I prevents glutamate-mediated bax translocation and cytochrome C release in O4+ oligodendrocyte progenitors. Glia (2004) 46(2):183–94. 10.1002/glia.10360 15042585

[58] Molinuevo JL Lladó A Rami L Memantine: targeting glutamate excitotoxicity in Alzheimer’s disease and other dementias. Am J Alzheimers Dis Other Demen (2005) 20(2):77–85. 10.1177/153331750502000206 15844753 PMC10833270

[59] Kumar A Singh RL Babu GN. Cell death mechanisms in the early stages of acute glutamate neurotoxicity. Neurosci Res (2010) 66(3):271–8. 10.1016/j.neures.2009.11.009 19944120

[60] Hota SK Barhwal K Baitharu I Prasad D Singh SB Ilavazhagan G. Bacopa monniera leaf extract ameliorates hypobaric hypoxia induced spatial memory impairment. Neurobiol Dis (2009) 34(1):23–39. 10.1016/j.nbd.2008.12.006 19154788

[61] Dief AE Kamha ES Baraka AM Elshorbagy AK. Monosodium glutamate neurotoxicity increases beta amyloid in the rat hippocampus: a potential role for cyclic AMP protein kinase. Neurotoxicology (2014) 42:76–82. 10.1016/j.neuro.2014.04.003 24769037

[62] Li Y Dai YB Sun JY Xiang Y Yang J Dai SY Neuroglobin attenuates beta amyloid-induced apoptosis through inhibiting caspases activity by activating PI3K/Akt signaling pathway. J Mol Neurosci (2016) 58(1):28–38. 10.1007/s12031-015-0645-z 26346601

[63] Jalali MR Roghani M The effect of *Nigella sativa* on learning and memory in male diabetic rats. (2009) 1(1):32–4.

[64] Salehi P Nasri S Roghani M Poordahandeh U Baluchnejadmojarad T The effect of thymoquinone on short-term spatial memory, passive avoidance learning and memory of diabetic rats and the involvement of hippocampal oxidative stress. Pajoohandeh J (2012) 17(5):219–27.

[65] Sahak MKA Mohamed AM Hashim NH Hasan Adli DS. *Nigella sativa* oil enhances the spatial working memory performance of rats on a radial arm maze. Evid Based Complement Alternat Med (2013) 2013:180598. 10.1155/2013/180598 24454487 PMC3888712

[66] Hosseini M Mohammadpour T Karami R Rajaei Z Reza Sadeghnia H Soukhtanloo M Effects of the hydro-alcoholic extract of *Nigella sativa* on scopolamine-induced spatial memory impairment in rats and its possible mechanism. Chin J Integr Med (2014) 21(6):438–44. 10.1007/s11655-014-1742-5 24584756

[67] Azzubaidi MS Saxena AK Talib NA Ahmed QU Dogarai BB. Protective effect of treatment with black cumin oil on spatial cognitive functions of rats that suffered global cerebrovascular hypoperfusion. Acta Neurobiol Exp (Wars) (2012) 72(2):154–65. 22810217 10.55782/ane-2012-1888

[68] Shao Y Feng Y Xie Y Luo Q Chen L Li B Protective effects of thymoquinone against convulsant activity induced by lithium-pilocarpine in a model of status epilepticus. Neurochem Res (2016) 41(12):3399–406. 10.1007/s11064-016-2074-y 27752802

[69] Kanter M. *Nigella sativa* and derived thymoquinone prevents hippocampal neurodegeneration after chronic toluene exposure in rats. Neurochem Res (2008) 33(3):579–88. 10.1007/s11064-007-9481-z 17929168

[70] Meral I Esrefoglu M Dar K Ustunova S Aydin M Demirtas M Effects of *Nigella sativa* on apoptosis and GABA_A_ receptor density in cerebral cortical and hippocampal neurons in pentylenetetrazol induced kindling in rats. Biotech Histochem (2016) 91(8):493–500. 10.1080/10520295.2016.1245866 27849392

[71] Ismail N Ismail M Azmi NH Bakar MFA Yida Z Abdullah MA Thymoquinone-rich fraction nanoemulsion (TQRFNE) decreases Aβ40 and Aβ42 levels by modulating APP processing, up-regulating IDE and LRP1, and down-regulating BACE1 and RAGE in response to high fat/cholesterol diet-induced rats. Biomed Pharmacother (2017) 95:780–8. 10.1016/j.biopha.2017.08.074 28892789

[72] Norsharina I Maznah I Iqbal S Latiff LA Anti-aggregation effects of thymoquinone against Alzheimers-amyloid in vitro. J Med Plant Res (2013) 7:31. Academic Journals, 2280–8. 10.5897/JMPR10.852

